# Supplementary material for: The Systems Biology Research Tool: evolvable open-source software
Source: BMC Syst Biol. 2008 Jun 29;2:55. doi: 10.1186/1752-0509-2-55 (PMC2446383; doi:10.1186/1752-0509-2-55)
Supplement: Additional file 1 — SBRT Archive. An archive of the current version of the Systems Biology Research Tool. [file 1752-0509-2-55-S1.zip › sbrt-1.4.0/doc/developers_guide/api/constant-values.html]

Constant Field Values


|  |  |  |
| --- | --- | --- |
| **Overview** \| Package \| Class \| Use \| **Tree** \| **Deprecated** \| **Index** \| **Help** | |  |
| PREV   NEXT | **FRAMES**    **NO FRAMES**     **All Classes** |  |


---


# Constant Field Values


---

**Contents**

- sbrt.\*

sbrt.\*

| sbrt.ProcessRunner | | |
| --- | --- | --- |
| `public static final java.lang.String` | `ERROR_LOG` | `"sbrt_error_log.txt"` |

| sbrt.Sbrt | | |
| --- | --- | --- |
| `public static final java.lang.String` | `GUI_ARG` | `"-g"` |
| `public static final int` | `MAJOR` | `1` |
| `public static final int` | `MINOR` | `4` |
| `public static final int` | `REVISION` | `0` |
| `public static final java.lang.String` | `USAGE` | `"usage: sbrt process_file.txt"` |
| `public static final java.lang.String` | `VERSION_ARG` | `"-v"` |

sbrt.kernel.\*

| sbrt.kernel.fba.comp.AbstractFluxIntervalComputer | | |
| --- | --- | --- |
| `public static final double` | `ZERO_CUTOFF` | `1.0E-9` |

| sbrt.kernel.fba.comp.CdHarFluxVectorComputer | | |
| --- | --- | --- |
| `public static final double` | `DEFAULT_TOLERANCE` | `1.0E-9` |

| sbrt.kernel.fba.comp.FbaOptComputer | | |
| --- | --- | --- |
| `public static final double` | `DEFAULT_CONST_TOL` | `0.0` |
| `public static final int` | `DEFAULT_SAFETY_LEVEL` | `1` |

sbrt.shell.\*

| sbrt.shell.ProcessManagerFactory | | |
| --- | --- | --- |
| `public static final java.lang.String` | `PROCESS` | `"Process"` |
| `public static final java.lang.String` | `PROCESS_NAME_FILE` | `"Process Name File"` |

| sbrt.shell.ProgramSolverFactory | | |
| --- | --- | --- |
| `public static final java.lang.String` | `CPLEX` | `"CPLEX"` |
| `public static final java.lang.String` | `GLPK` | `"GLPK"` |
| `public static final java.lang.String` | `PARAM_FILE` | `"Program Solver Parameter File"` |
| `public static final java.lang.String` | `SOLVER` | `"Program Solver"` |

| sbrt.shell.mng.AbstractProcessManager | | |
| --- | --- | --- |
| `public static final java.lang.String` | `ALGORITHM` | `"Algorithm"` |
| `public static final java.lang.String` | `COMPLETED` | `"Completed: "` |
| `public static final java.lang.String` | `DATA_HEADERS` | `"Data Headers"` |
| `public static final java.lang.String` | `ELAPSED_TIME` | `"Elapsed time: "` |
| `public static final java.lang.String` | `FILE_FORMAT` | `"File Format"` |
| `public static final java.lang.String` | `INPUT_FILE` | `"Input File"` |
| `public static final java.lang.String` | `INPUT_FILE_FORMAT` | `"Input File Format"` |
| `public static final java.lang.String` | `INPUT_FILE_NAME_FILE` | `"Input File Name File"` |
| `public static final java.lang.String` | `ITERATIONS` | `"Iterations"` |
| `public static final java.lang.String` | `OUTPUT_FILE_FORMAT` | `"Output File Format"` |
| `public static final java.lang.String` | `OUTPUT_FILE_NAME` | `"Output File Name"` |
| `public static final java.lang.String` | `OUTPUT_FILE_NAME_FILE` | `"Output File Name File"` |
| `public static final java.lang.String` | `PERCENT_COMPLETED` | `"Percent completed: "` |
| `public static final java.lang.String` | `SEED` | `"Seed"` |
| `public static final java.lang.String` | `ZERO_CUTOFF` | `"Zero Cutoff"` |

| sbrt.shell.mng.algebra.LinearSolverManager | | |
| --- | --- | --- |
| `public static final java.lang.String` | `EQUATIONS_FILE` | `"Equations File"` |
| `public static final java.lang.String` | `KERNEL_LINK_CMD` | `"Kernel Link Command"` |

| sbrt.shell.mng.comb.SingleElementUnionManager | | |
| --- | --- | --- |
| `public static final java.lang.String` | `SETS_FILE` | `"Sets File"` |

| sbrt.shell.mng.fba.BiggSbmlReaderManager | | |
| --- | --- | --- |
| `public static final java.lang.String` | `GENE_FILE_NAME` | `"Gene File Name"` |
| `public static final java.lang.String` | `RXN_FILE_NAME` | `"Reaction File Name"` |
| `public static final java.lang.String` | `SBML_INPUT_FILE` | `"SBML Input File"` |

| sbrt.shell.mng.fba.BiggSbmlTranslaterManager | | |
| --- | --- | --- |
| `public static final java.lang.String` | `GENE_FILE_NAME` | `"Gene File Name"` |
| `public static final java.lang.String` | `RXN_FILE_NAME` | `"Reaction File Name"` |
| `public static final java.lang.String` | `SBML_INPUT_FILE` | `"SBML Input File"` |

| sbrt.shell.mng.fba.CatalystDeletionManager | | |
| --- | --- | --- |
| `public static final java.lang.String` | `CATALYST_DELETION_FILE` | `"Catalyst Deletion File"` |

| sbrt.shell.mng.fba.FbaManager | | |
| --- | --- | --- |
| `public static final java.lang.String` | `CONSTRAINT_TOLERANCE` | `"Constraint Tolerance"` |
| `public static final double` | `DEFAULT_CONSTRAINT_TOLERANCE` | `1.0E-9` |
| `public static final java.lang.String` | `FLUX_VECTOR_FILE` | `"Flux Vector File"` |
| `public static final java.lang.String` | `FLUX_VECTOR_FILE_NAME` | `"Flux Vector File Name"` |
| `public static final java.lang.String` | `RXN_CATALYST_FILE` | `"Reaction-Catalyst File"` |
| `public static final java.lang.String` | `RXN_FILE` | `"Reaction File"` |
| `public static final java.lang.String` | `SYSTEM_SOLUTION_FILE` | `"System Solution File"` |

| sbrt.shell.mng.fba.FbaOptManager | | |
| --- | --- | --- |
| `public static final java.lang.String` | `CONSTRAINTS_FILE` | `"Constraints File"` |
| `public static final int` | `DEFAULT_SAFETY_LEVEL` | `1` |
| `public static final java.lang.String` | `OBJECTIVE_FUNCTION` | `"Objective Function"` |
| `public static final java.lang.String` | `OPTIMIZATION_SENSE` | `"Optimization Sense"` |
| `public static final java.lang.String` | `SAFETY_LEVEL` | `"Safety Level"` |

| sbrt.shell.mng.fba.FluxActivityManager | | |
| --- | --- | --- |
| `public static final java.lang.String` | `ACTIVITY_COUNT_FILE_NAME` | `"Activity Count File Name"` |
| `public static final java.lang.String` | `ACTIVITY_VECTOR_FILE_NAME` | `"Activity Vector File Name"` |
| `public static final java.lang.String` | `FLUX_VECTOR_FILE_FORMAT` | `"Flux Vector File Format"` |

| sbrt.shell.mng.fba.FluxCapManager | | |
| --- | --- | --- |
| `public static final java.lang.String` | `CYCLE_FILE` | `"Cycle File"` |

| sbrt.shell.mng.fba.FluxPlasticityManager | | |
| --- | --- | --- |
| `public static final java.lang.String` | `ALWAYS_ACTIVE_FILE_NAME` | `"Always-Active Reaction File Name"` |
| `public static final java.lang.String` | `FLUX_INTERVAL_FILE` | `"Flux Interval File"` |
| `public static final java.lang.String` | `NEVER_ACTIVE_FILE_NAME` | `"Never-Active Reaction File Name"` |
| `public static final java.lang.String` | `POT_ACTIVE_FILE_NAME` | `"Potentially-Active Reaction File Name"` |

| sbrt.shell.mng.fba.InitialPointManager | | |
| --- | --- | --- |
| `public static final double` | `DEFAULT_P` | `1.0E15` |
| `public static final java.lang.String` | `FLUX_INTERVAL_FILE` | `"Flux Interval File"` |
| `public static final java.lang.String` | `P_MAX` | `"P Max"` |
| `public static final java.lang.String` | `P_VECTOR_FILE_NAME` | `"P Vector File Name"` |

| sbrt.shell.mng.fba.IntervalVariationManager | | |
| --- | --- | --- |
| `public static final java.lang.String` | `CV_FILE` | `"Constraint Variation File"` |
| `public static final java.lang.String` | `FLUX_INTERVAL_FILE` | `"Flux Interval File"` |

| sbrt.shell.mng.fba.MmaNotebookManager | | |
| --- | --- | --- |
| `public static final java.lang.String` | `MMA_NOTEBOOK_NAME` | `"Mathematica Notebook Name"` |
| `public static final java.lang.String` | `SOLUTION_FILE_NAME` | `"Solution File Name"` |

| sbrt.shell.mng.fba.MsFluxIntervalManager | | |
| --- | --- | --- |
| `public static final java.lang.String` | `FLUX_CAP_FILE` | `"Flux Cap File"` |

| sbrt.shell.mng.fba.NetworkInfoManager | | |
| --- | --- | --- |
| `public static final java.lang.String` | `CATALYST_FILE_NAME` | `"Catalyst File Name"` |
| `public static final java.lang.String` | `CHEM_SPECIES_FILE_NAME` | `"Chemical Species File Name"` |
| `public static final java.lang.String` | `EQUATION_FILE_NAME` | `"Equation File Name"` |
| `public static final java.lang.String` | `EQUIV_RXN_FILE_NAME` | `"Equivalent Reaction File Name"` |
| `public static final java.lang.String` | `MATRIX_FILE_NAME` | `"Stoichiometry Matrix File Name"` |
| `public static final java.lang.String` | `REMOVE_REDUNDANT_REACTIONS` | `"Remove Redundant Reactions"` |
| `public static final java.lang.String` | `RXN_NAME_FILE_NAME` | `"Reaction Name File Name"` |
| `public static final java.lang.String` | `RXN_OUTPUT_FILE_NAME` | `"Reaction Output File Name"` |

| sbrt.shell.mng.fba.ObjectiveFunctionManager | | |
| --- | --- | --- |
| `public static final java.lang.String` | `OBJ_FUNCT_FILE` | `"Objective Function File"` |
| `public static final java.lang.String` | `OBJ_FUNCT_FILE_FORMAT` | `"Objective Function File Format"` |

| sbrt.shell.mng.fba.PalssonSbmlReaderManager | | |
| --- | --- | --- |
| `public static final java.lang.String` | `FLUX_BOUNDS_FILE_NAME` | `"Flux Bounds File Name"` |
| `public static final java.lang.String` | `FLUX_VECTOR_FILE_NAME` | `"Flux Vector File Name"` |
| `public static final java.lang.String` | `GENE_FILE_NAME` | `"Gene File Name"` |
| `public static final java.lang.String` | `PROTEIN_FILE_NAME` | `"Protein File Name"` |
| `public static final java.lang.String` | `RXN_FILE_NAME` | `"Reaction File Name"` |
| `public static final java.lang.String` | `SBML_INPUT_FILE` | `"SBML Input File"` |

| sbrt.shell.mng.fba.PalssonSbmlTranslaterManager | | |
| --- | --- | --- |
| `public static final java.lang.String` | `GENE_FILE_NAME` | `"Gene File Name"` |
| `public static final java.lang.String` | `PROTEIN_FILE_NAME` | `"Protein File Name"` |
| `public static final java.lang.String` | `RXN_FILE_NAME` | `"Reaction File Name"` |
| `public static final java.lang.String` | `SBML_INPUT_FILE` | `"SBML Input File"` |

| sbrt.shell.mng.fba.RandomConstraintsManager | | |
| --- | --- | --- |
| `public static final java.lang.String` | `ACTIVE` | `"Active Constraint"` |
| `public static final java.lang.String` | `INACTIVE` | `"Inactive Constraint"` |
| `public static final java.lang.String` | `NUMBER_OF_ON_FLUXES` | `"Number of Active Fluxes"` |

| sbrt.shell.mng.fba.RandomFluxVectorManager | | |
| --- | --- | --- |
| `public static final java.lang.String` | `ALGORITHM` | `"Random Flux Vector Algorithm"` |

| sbrt.shell.mng.fba.RxnDeletionManager | | |
| --- | --- | --- |
| `public static final java.lang.String` | `RXN_DELETION_FILE` | `"Reaction Deletion File"` |

| sbrt.shell.mng.fba.RxnRemovalManager | | |
| --- | --- | --- |
| `public static final java.lang.String` | `RXN_NAME_FILE` | `"Reaction Name File"` |

| sbrt.shell.mng.fba.SbmlWriterManager | | |
| --- | --- | --- |
| `public static final java.lang.String` | `SBML_OUTPUT_FILE_NAME` | `"SBML Output File Name"` |

| sbrt.shell.mng.fba.SimpleRxnFileTransManager | | |
| --- | --- | --- |
| `public static final java.lang.String` | `INPUT_RXN_FILE` | `"Input Reaction File"` |

| sbrt.shell.mng.fba.StoichVariationManager | | |
| --- | --- | --- |
| `public static final java.lang.String` | `MATRIX_FILE_NAMES_FILE` | `"Matrix File Names File"` |

| sbrt.shell.mng.fba.SvCdHarFluxVectorManager | | |
| --- | --- | --- |
| `public static final java.lang.String` | `OBSTRUCTED_MOVES_FILE_NAME` | `"Obstructed Moves File Name"` |

| sbrt.shell.mng.fba.SvInitialPointManager | | |
| --- | --- | --- |
| `public static final java.lang.String` | `AVG_P_FILE_NAME` | `"Average P File Name"` |

| sbrt.shell.mng.geom.CdHarManager | | |
| --- | --- | --- |
| `public static final java.lang.String` | `CONSTRAINT_TOLERANCE` | `"Constraint Tolerance"` |
| `public static final double` | `DEFAULT_CONSTRAINT_TOLERANCE` | `1.0E-9` |
| `public static final double` | `DEFAULT_MIN_CHORD_LENGTH` | `1.0E-6` |
| `public static final java.lang.String` | `INITIAL_POINT_FILE` | `"Initial Point File"` |
| `public static final java.lang.String` | `MAX_OBSTRUCTED_MOVES` | `"Maximum Obstructed Moves"` |
| `public static final java.lang.String` | `MIN_CHORD_LENGTH` | `"Minimum Chord Length"` |
| `public static final java.lang.String` | `PLANES_FILE` | `"Parallel Hyperplanes File"` |
| `public static final java.lang.String` | `POINTS_PER_POINT` | `"Points Per Point"` |

| sbrt.shell.mng.graph\_theory.CycleIdManager | | |
| --- | --- | --- |
| `public static final java.lang.String` | `EDGE_FILE` | `"Edge File"` |

| sbrt.shell.mng.graph\_theory.PathIdManager | | |
| --- | --- | --- |
| `public static final java.lang.String` | `EDGE_FILE` | `"Edge File"` |

| sbrt.shell.mng.stat.CorrelationManager | | |
| --- | --- | --- |
| `public static final java.lang.String` | `CORR_COEFF` | `"Correlation Coefficient"` |
| `public static final java.lang.String` | `KENDALLS_TAU` | `"Kendall\'s tau"` |
| `public static final java.lang.String` | `PEARSONS_R` | `"Pearson\'s r"` |
| `public static final java.lang.String` | `SPEARMANS_RHO` | `"Spearman\'s rho"` |
| `public static final java.lang.String` | `X_FILE_NAME` | `"X Value File Name"` |
| `public static final java.lang.String` | `Y_FILE_NAME` | `"Y Value File Name"` |

| sbrt.shell.mng.stat.KendallTauManager | | |
| --- | --- | --- |
| `public static final java.lang.String` | `TAU_A` | `"Kendall\'s Tau A"` |
| `public static final java.lang.String` | `TAU_B` | `"Kendall\'s Tau B"` |

| sbrt.shell.mng.stat.MannWhitneyUManager | | |
| --- | --- | --- |
| `public static final java.lang.String` | `SAMPLE_A_FILE` | `"Sample A File"` |
| `public static final java.lang.String` | `SAMPLE_B_FILE` | `"Sample B File"` |

| sbrt.shell.mng.utilities.IntervalComparisonManager | | |
| --- | --- | --- |
| `public static final java.lang.String` | `FILE_1` | `"File 1"` |
| `public static final java.lang.String` | `FILE_2` | `"File 2"` |

| sbrt.shell.mng.utilities.VariableParticipationManager | | |
| --- | --- | --- |
| `public static final java.lang.String` | `EXPRESSION_FILE` | `"Expression File"` |

| sbrt.shell.mng.utilities.VectorComparisonManager | | |
| --- | --- | --- |
| `public static final java.lang.String` | `FILE` | `"File"` |

| sbrt.shell.text.SpecialChars | | |
| --- | --- | --- |
| `public static final char` | `AMPERSAND` | `38` |
| `public static final char` | `ASTERICK` | `42` |
| `public static final char` | `AT` | `64` |
| `public static final char` | `BACK_SLASH` | `92` |
| `public static final char` | `CARET` | `94` |
| `public static final char` | `COLON` | `58` |
| `public static final char` | `COMMA` | `44` |
| `public static final char` | `DASH` | `45` |
| `public static final char` | `DOLLAR` | `36` |
| `public static final char` | `DOT` | `46` |
| `public static final char` | `EQUALS` | `61` |
| `public static final char` | `EXCLAMATION` | `33` |
| `public static final char` | `FORWARD_SLASH` | `47` |
| `public static final char` | `GREATER_THAN` | `62` |
| `public static final char` | `HASH` | `35` |
| `public static final char` | `LEFT_BRACE` | `123` |
| `public static final char` | `LEFT_BRACKET` | `91` |
| `public static final char` | `LEFT_PAREN` | `40` |
| `public static final char` | `LESS_THAN` | `60` |
| `public static final char` | `PERCENT` | `37` |
| `public static final char` | `PIPE` | `124` |
| `public static final char` | `PLUS` | `43` |
| `public static final char` | `QUESTION` | `63` |
| `public static final char` | `RIGHT_BRACE` | `125` |
| `public static final char` | `RIGHT_BRACKET` | `93` |
| `public static final char` | `RIGHT_PAREN` | `41` |
| `public static final char` | `SEMICOLON` | `59` |
| `public static final char` | `TILDE` | `126` |

| sbrt.shell.text.chem.IrrevRxnFormatV1 | | |
| --- | --- | --- |
| `public static final java.lang.String` | `ARROW` | `"-->"` |

| sbrt.shell.text.chem.IrrevRxnFormatV2 | | |
| --- | --- | --- |
| `public static final java.lang.String` | `ARROW` | `"-->"` |

| sbrt.shell.text.chem.RevRxnFormatV1 | | |
| --- | --- | --- |
| `public static final java.lang.String` | `ARROW` | `"<==>"` |

| sbrt.shell.text.chem.RevRxnFormatV2 | | |
| --- | --- | --- |
| `public static final java.lang.String` | `ARROW` | `"<==>"` |

| sbrt.shell.text.fba.BiggRxnNodeParser | | |
| --- | --- | --- |
| `public static final java.lang.String` | `RXN_NODE_NAME` | `"reaction"` |

| sbrt.shell.text.fba.FbaOptHeaderFormatterV1 | | |
| --- | --- | --- |
| `public static final java.lang.String` | `OBJ_VALUE` | `"Objective_Value"` |

| sbrt.shell.text.fba.FbaOptHeaderParserV1 | | |
| --- | --- | --- |
| `public static final java.lang.String` | `OBJ_VALUE` | `"Objective_Value"` |

| sbrt.shell.text.fba.IrrevRxnFormatV1 | | |
| --- | --- | --- |
| `public static final java.lang.String` | `ARROW` | `"-->"` |
| `public static final java.lang.String` | `SURR` | `"Surroundings"` |

| sbrt.shell.text.fba.PalssonRxnNodeParser | | |
| --- | --- | --- |
| `public static final java.lang.String` | `RXN_NODE_NAME` | `"reaction"` |

| sbrt.shell.text.fba.RxnNameSetFormatV1 | | |
| --- | --- | --- |
| `public static final java.lang.String` | `ALL_RXNS` | `"All_Reactions"` |

---


|  |  |  |
| --- | --- | --- |
| **Overview** \| Package \| Class \| Use \| **Tree** \| **Deprecated** \| **Index** \| **Help** | |  |
| PREV   NEXT | **FRAMES**    **NO FRAMES**     **All Classes** |  |


---
